# Development and validation of Nadamay meditation: A sound-based protocol for psychosocial well-being

**DOI:** 10.1016/j.jaim.2026.101375

**Published:** 2026-07-25

**Authors:** Km Megha, Arjun Ram Roj, Sanjib Patra

**Affiliations:** a Department of Yoga, Central University of Rajasthan, Ajmer, Rajasthan, 305817, India

**Keywords:** Nadamay meditation, Nada yoga, Psychosocial well-being, Meditation validation, Content validity, Mental health

## Abstract

**Background:**

Psychosocial well-being is increasingly recognized as a crucial aspect of overall health, with stress, anxiety, and emotional dysregulation affecting individuals across various demographics. Meditation has been widely studied for its potential to enhance mental well-being. This study developed and validated the Nadamay meditation technique, a structured practice based on ancient *Nada* yoga principles from the Nada Bindu Upanishad.

**Objective:**

To develop and validate the *Nadamay* meditation protocol, ensuring its comprehensiveness and content validity for promoting psychosocial well-being, including aspects of mental, emotional, and social health, across diverse populations.

**Methods:**

The *Nadamay* meditation protocol was developed by incorporating the principles of the *Nada Bindu* Upanishad. 21 subject matter experts from various fields reviewed the seven meditation steps using the Content Validity Ratio (CVR) and Item-level Content Validity Index (I-CVI) methods.

**Results:**

All seven steps demonstrated acceptable to excellent content validity, with CVR scores ranging from 0.52 to 1.0 and I-CVI scores from 0.76 to 1.0, exceeding critical thresholds. The Scale-level Content Validity Index Average (S-CVI/Ave) was 0.92, confirming excellent overall content validity and the protocol's comprehensiveness.

**Conclusion:**

The Nadamay meditation protocol is a structured, content-validated intervention for psychosocial well-being. While preliminary validation is promising, its inclusion of energizing practices like *Kapalbhati* and its broad design necessitate further feasibility and safety testing before widespread implementation.

## 1 Introduction

In today's fast-paced world, individuals across various demographics encounter numerous mental health challenges that adversely affect psychosocial well-being across all age and occupational groups [[1], [2], [3]]. Factors such as occupational stress, financial strain, social anxieties, and the complexities of modern life have been associated with elevated mental health risk [[1], [2], [3], [4]].

Meditation has emerged as a promising intervention for reducing stress and improving mental well-being. Systematic reviews have shown that meditative practices can reduce stress, improve attention, and foster emotional balance [[5], [6], [7]]. Among these, sound-based meditation techniques, including mantra, toning, and vibrational focus, have drawn increasing empirical interest [8,9]. However, despite the expanding body of literature on general sound meditation and *Nada* Yoga-inspired music therapies, a gap remains in structured, accessible, scripturally aligned meditation protocols specifically rooted in classical Nada Yoga texts and subjected to rigorous content validation.

One of its principal sources is the Nada Bindu Upanishad, which presents sound (Nada) as both the means and the object of meditation [10,11]. Verses 33-35 describe a practitioner's journey through progressively subtle inner sounds, from thunder and drums to bees and flutes, signifying the inward migration of awareness. Verse 39 likens absorption in Nada to the merging of milk into water, illustrating deep mental unification. Other verses (41, 44-45) highlight Nada as a restraining tool that stabilizes attention and leads toward unmani, a thoughtless state of pure awareness [10,11].

To adapt this framework for contemporary use, we developed Nadamay Meditation, a structured protocol consisting of seven sequential stages: (1) Prayer, (2) *Kapalbhati*, (3) Breath Awareness, (4) Contemplation on *Bhava* (Emotion), (5) Concentration on Sound (AUM), (6) Silence (*Ajapa Japa*), and (7) Closing Mantra (Shanti Mantra). This protocol mirrors the progression outlined in the Nada Bindu Upanishad, from external activity to internal stillness, while incorporating modern psychological concepts and modifications for accessibility.

Unlike traditional Nada Yoga approaches that rely on spontaneous internal sounds, *Nadamay* Meditation offers a time-bound, teacher-friendly framework for gradual internalization. This integration of structured breath, emotional reflection, and auditory immersion seeks to make Nada Yoga principles accessible for contemporary populations, including those with no prior meditation experience. By 'modern' or 'contemporary populations,' we refer to individuals across diverse age groups, socioeconomic backgrounds, educational levels, and cultural contexts who may have limited exposure to traditional yogic practices but seek accessible, evidence-based interventions for mental health and psychosocial well-being.

The present manuscript outlines the development and initial content validation of the Nadamay Meditation protocol using the Content Validity Ratio (CVR) and Content Validity Index (CVI) methods. By drawing on scriptural inspiration, expert feedback, and systematic design, this work aims to establish a theoretically grounded foundation for future empirical exploration into the psychological and physiological effects of sound-based meditative practices.

## 2 Methods

The development and validation of Nadamay meditation were conducted through two sequential phases.

### 2.1 Phase 1: development of *Nadamay* meditation

The development of the *Nadamay* meditation protocol began with a systematic literature review of both classical yogic texts and contemporary scientific literature, with a special focus on the Nada Bindu Upanishad. The aim was to identify core practices from *Nada* yoga that could be integrated into a structured and teachable meditation sequence to support psychosocial well-being.

The literature review was conducted between January 2000 and December 2023 using databases such as PubMed, Google Scholar, AYUSH Research Portal, Web of Science, Medline, and Scopus. Search terms included *Nada* yoga, sound meditation, Upanishad, OM meditation, Bhava, mindfulness, and yogic intervention validation. Inclusion criteria were primary or review articles describing theoretical models or empirical evidence related to sound-based meditative practices grounded in classical yoga traditions. Non-scriptural or purely instrumental sound therapy approaches without a yogic lineage were excluded.

The review was carried out by a team of five certified yoga researchers, each with over five years of teaching and scriptural study experience. Their inclusion was intentional to ensure that both traditional authenticity and instructional feasibility were incorporated during protocol design.

Based on the literature review and scriptural analysis, the Nadamay protocol was developed as a seven-step sequence (Table 1). Each step was selected based on its textual grounding in Nada yoga and its alignment with contemporary psychological frameworks. Opening prayer and closing mantras were included to establish psychological boundaries and facilitate transition, consistent with ritual framing in contemplative practices [12,13]. *Kapalbhati*, while not central to classical *Nada* yoga texts, was incorporated as a preparatory energizing practice based on evidence of its effects on alertness and respiratory function [14,15]. Breath awareness was included to develop interoceptive attention, a foundational skill in mindfulness-based interventions [16,17]. The *Bhava* (emotional contemplation) component draws on positive psychology and affective neuroscience research demonstrating the role of positive emotions in psychological well-being [18,19]. The core sound meditation phase (AUM chanting and listening) directly reflects Nada Bindu Upanishad verses 33-39, supported by neuroimaging studies of mantra meditation [20]. The final silent meditation phase (*Ajapa Japa*) represents the transition to internal subtle sound awareness (*Anahata Nada*), aligning with advanced *Nada* yoga practices described in verses 41-45 and research on meditative states [21,22].

**Table 1:** The Nadamay meditation protocol: Detailed description of seven sequential steps.

Step	Practice	Description and Rationale
1	Prayer (30 s)	Opening prayer or invocation to foster reverence, emotional readiness, and mental preparation. Creates a sacred space for practice and aligns intention.
2	*Kapalbhati* (1 min)	Energizing breathing technique (3 rounds of 30-50 breaths). While not central to Nada yoga scriptures, incorporated for its preparatory function, mental alertness, and physiological activation.
3	Breath Awareness (4 min)	Mindful observation of natural breathing patterns. Enables interoception and facilitates transition from gross sensation to subtle attention, preparing for deeper meditative states.
4	Contemplation on *Bhava* (6 min)	Cultivation of positive emotional states (compassion, gratitude, surrender). Though not a classical Nada practice, this step aligns with affective neuroscience and contemplative psychology models, serving as a bridge to auditory immersion.
5	Concentration on Sound (AUM) (15 min)	Focused chanting and listening to the AUM sound. Core practice of Nada Yoga, utilizing external sound (*Ahata Nada*) to direct attention inward and stabilize the mind.
6	Silence/*Ajapa Japa* (5 min)	Silent meditation attending to internal subtle sounds (*Anahata Nada*) or thoughtless awareness. Represents the culmination of the Nada yoga journey toward inner stillness and the unmani state.
7	*Shanti Mantra* (30 s)	Closing peace mantra for gradual transition back to normal awareness. Promotes integration of meditative experience and collective well-being.

Note: Total duration approximately 32 min.

The resulting seven-step protocol is presented in Table 1, which details each component's practice description and underlying rationale. The protocol begins with a brief opening prayer (30 s) to establish psychological readiness and ceremonial framing. This is followed by Kapalbhati (1 min), a preparatory breathing practice to energize and alert the system. Breath awareness (4 min) then cultivates interoceptive attention and calms the nervous system. Contemplation on *Bhava* (6 min) serves as an emotional preparation phase, fostering positive affective states conducive to meditative depth. The core practice consists of 15 min of AUM chanting and auditory focus, representing the central Nada yoga component of sound-based meditation. This transitions into 5 min of silent meditation (*Ajapa Japa*), where practitioners attend to internal subtle sounds or thoughtless awareness. The protocol concludes with a brief *Shanti Mantra* (30 s) for integration and closure. The total duration is approximately 32 min, designed to balance comprehensiveness with practical feasibility for daily practice.

### 2.2 Phase 2: content validation

Content validation was performed using the Content Validity Ratio (CVR) method developed by Lawshe [23] and the Content Validity Index (CVI) approach recommended by Polit and Beck [24]. A panel of twenty-one subject matter experts (SMEs) was assembled, representing diverse fields including yoga therapy (n = 7), psychology (n = 5), Ayurveda (n = 4), integrative medicine (n = 3), and meditation research (n = 2). The sample size of 21 experts was determined following content validation guidelines, which recommend a minimum of 6-10 experts for basic validation, with larger panels (≥20) preferred for comprehensive assessment and enhanced statistical power [24,25] [Lynn, 1986; Polit & Beck, 2006]. Each expert had a minimum of five years of professional experience in their respective domain, with the majority holding advanced degrees (PhD, MD, or equivalent) and active involvement in research or clinical practice. The characteristics of the expert panel are presented in Table 2.

**Table 2:** Characteristics of subject matter experts (N = 21).

Field of Expertise	Number (n)	Experience Range (years)	Highest Qualification
Yoga Therapy	7	5-15	PhD/MD
Psychology	5	6-18	PhD
Ayurveda	4	7-20	MD (Ayurveda)/PhD
Integrative Medicine	3	8-16	MD/PhD
Meditation Research	2	10-14	PhD
Total	21	5-20	-

Note: All experts had active involvement in research or clinical practice in their respective fields.

As shown in Table 2, the expert panel composition reflected diverse disciplinary perspectives essential for comprehensive protocol validation. The largest representation came from yoga therapy (n = 7, 33%), providing expertise in traditional yogic practices and their contemporary therapeutic applications. Psychology experts (n = 5, 24%) contributed insights into mental health frameworks and psychosocial well-being constructs. Ayurveda practitioners (n = 4, 19%) ensured alignment with traditional Indian healing systems and holistic health principles. Integrative medicine specialists (n = 3, 14%) offered perspectives on evidence-based complementary approaches, while meditation researchers (n = 2, 10%) provided methodological expertise in contemplative science. The panel's experience ranged from 5 to 20 years, with most holding doctoral-level qualifications (PhD or MD), ensuring both theoretical depth and practical knowledge. This multidisciplinary composition was intentional, designed to evaluate the protocol from multiple expert perspectives including traditional authenticity, psychological validity, clinical applicability, and research rigor.

The experts were provided with detailed descriptions of each meditation step (as shown in Table 1) and asked to rate whether each step was essential, useful but not essential, or not necessary for achieving the protocol's objectives of promoting psychosocial well-being.

The CVR was calculated using Lawshe's formula: CVR = (ne - N/2)/(N/2), where ne is the number of experts indicating 'essential' and N is the total number of experts. For 21 experts, the critical CVR value at p < 0.05 is 0.42 [26]. The Item-level Content Validity Index (I-CVI) was calculated as I-CVI = ne/N. An I-CVI of 0.78 or higher indicates excellent item-level content validity [24,25]. The Scale-level Content Validity Index Average (S-CVI/Ave) was computed as the average of all I-CVI values. An S-CVI/Ave of 0.90 or higher indicates excellent overall content validity [25,27].

## 3 Results

All seven steps demonstrated strong content validity, with CVR scores ranging from 0.52 to 1.0 and I-CVI scores ranging from 0.76 to 1.0 (Table 3), all exceeding critical thresholds. The S-CVI/Ave was 0.92, indicating excellent overall content validity. Examining Table 3 in detail reveals varying levels of expert consensus across protocol components. The highest consensus was achieved for Breath Awareness (Step 3) and Concentration on Sound/AUM (Step 5), both receiving unanimous expert endorsement (21/21, CVR = 1.0, I-CVI = 1.0). This reflects strong agreement on the centrality of mindful breathing and sound-based focus to the protocol's objectives. Steps 1 (Prayer), 6 (Silence/*Ajapa Japa*), and 7 (Shanti Mantra) also demonstrated very high consensus (20/21, CVR = 0.90, I-CVI = 0.95), indicating near-unanimous recognition of their importance for framing and deepening the meditative experience. The relatively lower ratings for Step 4 (Contemplation on Bhava: 16/21, CVR = 0.52, I-CVI = 0.76) and Step 2 (*Kapalbhati*: 17/21, CVR = 0.62, I-CVI = 0.81) suggest some expert variability regarding preparatory and emotional components. However, both still exceeded critical thresholds (CVR ≥0.42, I-CVI ≥0.78), supporting their retention. The overall S-CVI/Ave of 0.92 surpasses the 0.90 benchmark for excellent scale-level validity, confirming that the protocol as a whole is comprehensive, relevant, and well-designed for promoting psychosocial well-being.

**Table 3:** Content validity results for *Nadamay* meditation protocol (N = 21 experts).

Protocol Step	Experts Rating Essential	CVR	I-CVI	Decision
1. Prayer	20	0.9	0.95	Retained
2. *Kapalbhati*	17	0.62	0.81	Retained
3. Breath Awareness	21	1	1	Retained
4. Contemplation on *Bhava*	16	0.52	0.76	Retained
5. Concentration on Sound (AUM)	21	1	1	Retained
6. Silence/*Ajapa Japa*	20	0.9	0.95	Retained
7. *Shanti Mantra*	20	0.9	0.95	Retained
Overall Protocol (S-CVI/Ave)	-	-	0.92	Excellent

Note: CVR = Content Validity Ratio; I-CVI = Item-level Content Validity Index; S-CVI/Ave = Scale-level Content Validity Index Average. Critical values: CVR ≥0.42 (for N = 21, p < 0.05); I-CVI ≥0.78 (excellent); S-CVI/Ave ≥0.90 (excellent).

Qualitative feedback from experts emphasized the protocol's logical progression from preparatory practices to deep meditative states, its grounding in traditional *Nada* yoga philosophy, and its potential accessibility for contemporary practitioners.

## 4 Discussion

This study presents the development and preliminary content validation of the Nadamay Meditation protocol, a structured sound-based meditation technique rooted in classical Nada yoga principles. The high CVR and I-CVI scores across all seven steps, along with an excellent S-CVI/Ave of 0.92, confirm that the protocol is theoretically sound, comprehensive, and relevant for promoting psychosocial well-being. While content validity has been established, real-world feasibility and clinical effectiveness remain to be evaluated through empirical testing.

### 4.1 Integration of traditional and contemporary approaches

The integration of traditional Nada yoga principles with contemporary meditation structures represents a novel approach to making ancient contemplative practices accessible to modern populations. While the protocol draws heavily from the Nada Bindu Upanishad, it incorporates preparatory practices such as Kapalbhati and emotional contemplation (Bhava) that are not traditionally central to Nada yoga but serve important practical functions. The slightly lower I-CVI for Kapalbhati (0.76) reflects expert recognition that while beneficial for preparation, it is not essential to the core Nada yoga framework. Nevertheless, it exceeded the critical threshold, supporting its inclusion.

While Siddhasana and Vaishnavi Mudra are emphasized in traditional *Nada* yoga texts, they were intentionally excluded to enhance accessibility for individuals with physical limitations or those unfamiliar with classical postures, based on expert feedback and pilot observations. Flexible seating options (supported sitting, cross-legged) were encouraged to ensure broader usability without compromising meditative depth.

The protocol's design features several characteristics that enhance its practical applicability for promoting psychosocial well-being in real-world contexts. The structured sequential format allows for standardized teaching and systematic skill development, making it suitable for group instruction in mental health settings, workplace wellness initiatives, and community-based interventions. The 32-min duration strikes a balance between comprehensiveness and practicality, fitting within typical therapy session lengths or lunch-break wellness programs. The inclusion of preparatory practices (prayer, Kapalbhati, breath awareness) before deeper meditative components makes the protocol accessible to beginners while maintaining depth for experienced practitioners. The emphasis on sound-based meditation addresses multiple dimensions of psychosocial well-being: mental health through attention regulation and stress reduction, emotional health through positive affect cultivation (Bhava), and social health through potential group practice and shared contemplative experience. These features suggest applicability across diverse populations seeking non-pharmacological interventions for anxiety, stress, emotional dysregulation, and general mental wellness enhancement.

From a validation perspective, these results support the protocol's suitability for implementation across diverse settings where psychosocial well-being interventions are needed. The high content validity across all steps (S-CVI/Ave = 0.92) indicates the protocol addresses core components necessary for mental, emotional, and social health promotion. The unanimous expert endorsement of breath awareness and sound meditation components (CVR = 1.0) confirms these practices are universally recognized as essential for stress reduction and emotional regulation. The protocol's structured, time-bounded format (32 min) and sequential progression make it potentially applicable in workplace wellness programs, educational institutions, mental health clinics, community centers, and individual home practice. However, the lower consensus on *Kapalbhati* highlights the need for careful implementation guidance, particularly regarding contraindications and individual suitability assessment before widespread dissemination.

### 4.2 Theoretical mechanisms and Neuroscientific support

**Fig. 1:**
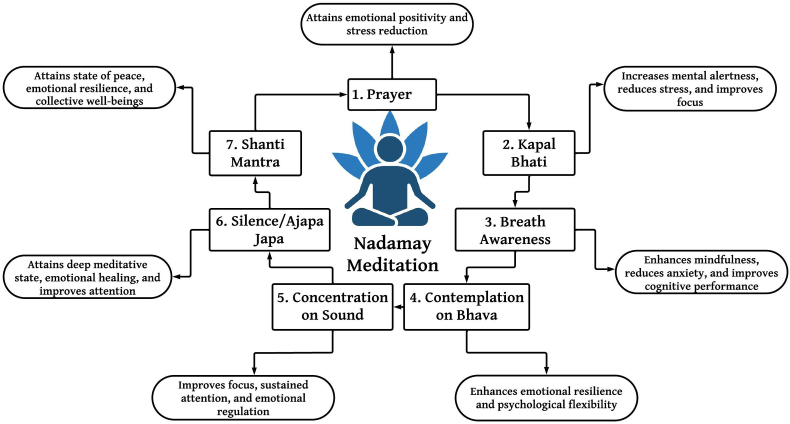
Conceptual Framework of the Nadamay Meditation Protocol. Note: The figure illustrates the conceptual framework of the Nadamay Meditation protocol, showing the seven sequential steps and their proposed psychosocial benefits.

The protocol's emphasis on sound-based meditation aligns with emerging research on neurophysiological effects of mantra and toning practices. Studies have shown that OM chanting is associated with deactivation of limbic brain regions involved in emotional processing [20], and that meditation practices can modulate brain activity patterns associated with relaxation [28]. The sequential structure, progressing from external to internal auditory focus, is designed to facilitate gradual internalization of attention, supported by research on attention regulation in meditation [29]. The integration of emotional contemplation draws on positive emotion theory [30], serving as a psychological bridge to deeper meditative states. The proposed progression from preparatory practices through sound immersion to silent meditation, as illustrated in Fig. 1, reflects the traditional *Nada* yoga principle of transitioning from gross external stimuli to subtle internal awareness.

### 4.3 Limitations and future directions

Several limitations warrant consideration. This study focused solely on content validity; feasibility, acceptability, and efficacy remain untested. Future research should conduct feasibility studies with diverse participant populations. *Kapalbhati*, traditionally a cleansing kriya, may be contraindicated for individuals with hypertension, cardiovascular conditions, epilepsy, hernias, anxiety disorders, or during pregnancy. While it is limited to 1 min at low intensity in the protocol and positioned as optional preparatory practice, formal safety testing in these populations remains necessary. The protocol has not been tested across diverse demographic groups, and cultural adaptations may be necessary for different populations, particularly given the emphasis on Sanskrit terminology which may require modification for cross-cultural applicability. While the expert panel was professionally diverse, all experts were from India; international perspectives could provide insights into cross-cultural relevance. This study did not include construct validity, criterion validity, or reliability testing, and comprehensive psychometric validation is needed. Finally, the 32-min duration may be challenging for beginners or individuals with limited time, warranting exploration of abbreviated versions while maintaining theoretical integrity.

Additionally, real-world implementation challenges warrant consideration: the protocol requires trained facilitators familiar with both yogic principles and psychosocial health frameworks, appropriate quiet spaces for group or individual practice, and cultural sensitivity when introducing Sanskrit terminology and traditional practices to diverse populations. Practical barriers such as time constraints in clinical settings, participant motivation and adherence, and integration with existing mental health services will need to be addressed through feasibility studies and implementation science approaches before large-scale dissemination for psychosocial well-being promotion.

## 5 Conclusion

The Nadamay Meditation protocol represents a theoretically grounded, content-validated approach to sound-based meditation for psychosocial well-being. The protocol successfully integrates classical *Nada* yoga principles with contemporary frameworks, making it accessible while maintaining scriptural authenticity. Future research should focus on feasibility testing, safety evaluation regarding *Kapalbhati*, and efficacy trials using randomized controlled designs to assess impact on psychosocial outcomes.

## Author contributions

All authors contributed to study conception and design. Conceptualization, Methodology/Study design, Validation, Formal analysis, Data curation, Writing - original draft by [KM]. Conceptualization, Methodology/Study design, Visualization, Investigation by [ARR]. Writing - review and editing, Visualization, Supervision, Project administration.

All authors read and approved the final manuscript.

## Declaration of generative AI in scientific writing

None.

## Funding sources

None.

## Conflict of interest

The authors declare the following financial interests/personal relationships which may be considered as potential competing interests:Prof. Sanjib Patra reports administrative support was provided by Central University of Rajasthan. Sanjib Patra reports a relationship with Central University of Rajasthan that includes: employment. The authors declare no competing interests. There are no additional relationships or activities that could be perceived as potential conflicts of interest related to this work.

## Data Availability

Data are available from the corresponding author upon reasonable request.
